# COVID-19 and the Immune Response: A Multi-Phasic Approach to the Treatment of COVID-19

**DOI:** 10.3390/ijms23158606

**Published:** 2022-08-03

**Authors:** Tzuriel Sapir, Zaelig Averch, Brian Lerman, Abraham Bodzin, Yeshaya Fishman, Radhashree Maitra

**Affiliations:** Department of Biology, Yeshiva College, Yeshiva University, 500 W 185th Str., New York, NY 10033, USA; tsapir@mail.yu.edu (T.S.); averch@mail.yu.edu (Z.A.); balerman@mail.yu.edu (B.L.); bodzin@mail.yu.edu (A.B.); yfishma1@mail.yu.edu (Y.F.)

**Keywords:** COVID-19, SARS-CoV-2, multi-phasic, immune response, natural killer cell, cytokine storm, IL-6, treatment, monoclonal antibody, immunotherapy

## Abstract

Severe acute respiratory syndrome coronavirus 2 (SARS-CoV-2) is a viral agent that causes Coronavirus disease 2019 (COVID-19), a disease that causes flu-like symptoms that, when exacerbated, can have life-threatening consequences. COVID-19 has been linked to persistent symptoms, sequelae, and medical complications that can last months after the initial infection. This systematic review aims to elucidate the innate and adaptive immune mechanisms involved and identify potential characteristics of COVID-19 pathology that may increase symptom duration. We also describe he three different stages of COVID-19—viral replication, immune hyperactivation, and post-acute sequelae—as well as each phase’s corresponding immune response. Finally, we use this multiphasic approach to describe different treatment approaches for each of the three stages—antivirals, immunosuppressants and monoclonal antibodies, and continued immunosuppressants—to fully curate the treatment to the stage of disease.

## 1. Introduction

COVID-19, a viral infectious disease, is caused by the novel SARS-CoV-2 of the family Coronaviridae and genus Betacoronavirus [1]. As of July 2022, there have been an estimated 573 million cases of COVID-19 and more than 6.4 million deaths [2]. Most patients with COVID-19 experience minimal flu-like symptoms, or even none at all. However, approximately one fifth of COVID-19 patients experience severe disease or die [3]. Underlying medical conditions and advanced age are known to be risk factors for severe COVID-19 [4].

**Figure 1:**
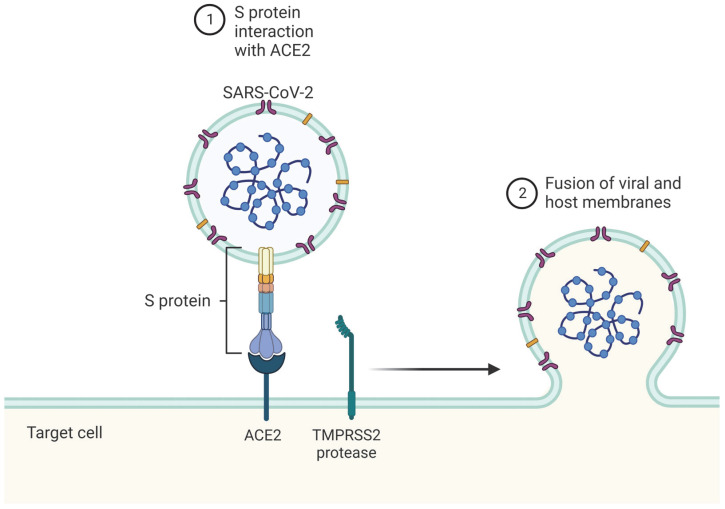
(1) SARS-CoV-2 uses its spike (S) protein to bind host angiotensin-converting enzyme 2 (ACE2). (2) Transmembrane serine protease 2 (TMPRSS2), a second host membrane protein, cleaves S protein to initiate the fusion of viral and host membrane. Thus, viral tropism of SARS-CoV-2 is highly determined by the presence of both ACE2 and TMPRSS2 on the host membrane.

SARS-CoV-2 is similar to other coronaviruses in that it is an enveloped, spherical virus with a single-stranded, positive-sense RNA genome. Therefore, the entire viral life cycle takes place within the host cell which has been infected. The virus enters the host cell by using its viral spike (S) glycoprotein to interact with angiotensin-converting enzyme 2 (ACE2), a host receptor protein [1]. Transmembrane serine protease 2 (TMPRSS2), the second protein involved in this entry process, facilitates the fusion of viral and host membranes, and allows the virus to be released into the host cytoplasm (Figure 1). As such, viral tropism of SARS-CoV-2 is determined by the presence of ACE2 and TMPRSS2 on the host cell’s plasma membrane, meaning that tissues such as nasal epithelial cells, lungs, and bronchial branches—where co-expression of both is high—are affected most [3,5,6].

The incubation period between SARS-CoV-2 exposure and symptom onset is about five days, although this may vary [7]. COVID-19 infection is known to be multi-phasic, meaning that there are several stages to the infection process. This makes patients with severe COVID-19 infection difficult to treat, as different therapeutic approaches are required depending on the patient’s stage of infection [8]. For example, the first phase of COVID-19 infection is a viral replication phase during which it is most beneficial to use drugs that inhibit viral replication. However, during the subsequent phase of inflammation, during which an overwhelming and potentially harmful immune response takes place, only drugs that reduce the immune response will be helpful [9]. For this reason, it is important to understand the immune system’s full breadth of response to COVID-19 infection.

The first phase of infection involves a viral replication phase. In this phase, SARS-CoV-2 establishes itself in the host body and proceeds to rapidly duplicate itself. In the following two sections we describe the ways in which the innate and adaptive immune responses behave during this stage. We delineate the ways the virus escapes immunity and describe how the initial immune response brings about the second stage of infection, that of immune hyperactivation. The fourth section of this paper looks at that second phase alongside viral clearance, while the fifth section explores a phenomenon known as Post-Acute Sequelae of COVID-19—a proposed third stage of COVID-19 infection. Finally, the sixth section of our review details current treatment practices for COVID-19. An overview of the multiple stages of infection and their treatments are illustrated in Table 1.

## 2. Innate Immune Response

### 2.1. Complement Activation

The body’s most immediate line of defense, the complement system, is usually vital for a quick and effective immune response, but in coronaviruses may sometimes cause more harm than good. While complement activation during the first week after infection can successfully fight COVID-19, prolonged complement activation can lead to a positive feedback loop in inflammation that contributes to multi-organ failure in severe cases of COVID-19 [16]. In mouse studies of the closely related SARS-CoV, it was found that products of C3 activation such as C3a, C3b, and iC3b were detectable in the lungs a single day after infection, and that C3^−/−^ mice had less severe lung injury, had fewer neutrophils and inflammatory monocytes, and had reduced cytokine and chemokine levels. Additionally, C3^−/−^, factor B^−/−^, and C4^−/−^ mice lost less weight over the course of infection than wild type mice [17]. Furthermore, experiments blocking C3 or C5 have been found to reduce disease severity, respiratory impairment, and cytokine response [18].

Recently, it has also been reported that SARS-CoV-2 nucleoprotein dimers activate mannose-binding protein-associated serine protease 2 (MASP-2), which is the main trigger for activation of the lectin pathway of the complement system. This in turn yields C3 convertase and the membrane attack complex [19]. Conversely, suppressing either the nucleoprotein MASP-2 interaction or complement activation led to less lung injury [19]. SARS-CoV-2 thus stimulates the complement system, resulting in extended complement activation and inflammation.

These findings are further substantiated. For example, patients with macular degeneration, a complement-mediated disease, were found to have a much higher risk of developing severe COVID-19, suggesting a possible connection between the increased presence of complement proteins and worse COVID-19 outcome [20]. Additionally, patients with COVID-19 are found to have raised levels of complement proteins in their plasma and complement fragment deposition in certain organs [21,22]. They are also found to experience neutrophilia, the over-abundance of neutrophils in the blood, to the extent that the neutrophil: leukocyte ratio has been shown to be an independent risk factor for serious COVID-19 [23,24]. Activated neutrophils and neutrophil extracellular traps (NETs) contain complement proteins necessary for the alternative C3 convertase, providing yet another way for COVID-19 to induce prolonged complement activation [16,25].

### 2.2. Immune Detection of SARS-CoV-2

Both the absence of, and interference with, toll-like receptor 2 (TLR2) has been shown to decrease pro-inflammatory response to SARS-CoV-2, suggesting that TLR2 is one of the pattern recognition receptors (PRRs) that recognize the virus. Specifically, inhibition of TLR2 decreased inflammatory response after it had been induced with SARS-CoV-2 envelope protein, suggesting that envelope protein may be that which TLR2 recognizes on SARS-CoV-2 [26]. Other TLRs are less closely studied in the context of SARS-CoV-2. TLR3, which recognizes double-stranded RNA (dsRNA), has been shown to be activated by SARS-CoV-2 infection within the first 24 h [27]. TLR1, TLR4, and TLR6 may bind SARS-CoV-2 spike protein [28]. However, because S protein has been shown to preferentially bind lipopolysaccharide, a known target of TLR4, the possibility of lipopolysaccharide contamination of S protein causing a TLR4-mediated cytokine reaction has cast some doubt over these findings [29]. TLR7 has also been shown to activate in response to SARS-CoV-2, and abnormalities in the TLR7 gene correlate with severe COVID-19 [27,30]. Table 2 described the TLRs used to detect the presence of SARS-CoV-2.

### 2.3. SARS-CoV-2 Avoids Innate Immunity

SARS-CoV-2 has strategies to avoid being detected or acted upon by the immune system. Several studies have together shown that the SARS-CoV-2 immune evasion strategy involves restricting the interferon (IFN) system, resulting in low type I and II IFN responses, as well as low IFN-stimulated genes (ISGs) during the early stages of COVID-19 [31,32]. The SARS-CoV-2 proteins responsible for this include non-structural protein 1 (NSP1), NSP8, NSP9, NSP13, NSP15, ORF9b, and ORF6 [33,34,35]. This provides for SARS-CoV-2 to establish itself in the body without as much a threat from early immune response.

### 2.4. IFN and IL Response

Similar to what has been suggested in the complement system, the induced innate immune response to COVID-19 is both necessary for effective disease suppression, as in other diseases, and capable of causing severe damage to the host. In their intended role, IFNs help clear infection from the host body by promoting the production of antiviral compounds by transcription of ISGs and cytokines. However, in severe COVID-19, positive feedback loops in cytokines and IFNs can lead to cytokine storm—the dysregulated release of cytokines—leading to hyperinflammation, multiorgan failure, and death.

**Figure 2:**
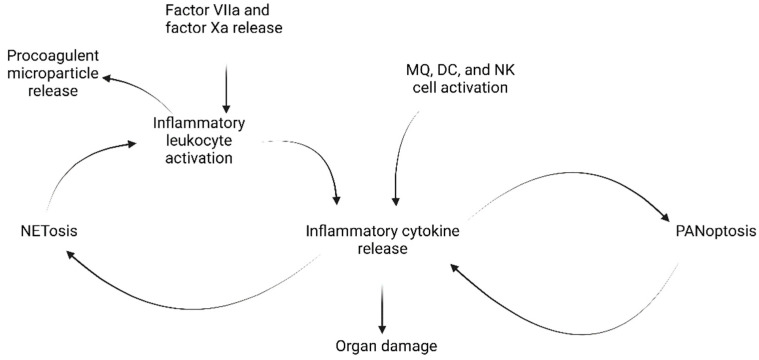
Inflammatory cytokines are present in both cytokine storm and procoagulant feedback loops.

Upon detection by PRRs, immune cells such as macrophages, dendritic cells, and natural killer cells (NK cells) release IFNs and proinflammatory cytokines including IL-1β, IL-6, TNF-α, IL-12, and IFN-γ [11]. TNF-α and IFN-γ together stimulate PANoptosis—an innate immune programmed cell death pathway separate from apoptosis, pyroptosis (inflammatory programmed cell death), or necrosis (programmed cell death by necrosis)—which in turn stimulates further proinflammatory cytokine release, resulting in cytokine storm (Figure 2) [36]. The prolonged inflammation and associated endothelial cell damage can eventually contribute to symptoms of severe COVID-19 such as lung damage, acute respiratory distress syndrome, organ failure, or even death [37]. This pathway is especially seen in the second week after disease onset, following the decline in IFN seen in the earliest phase of disease. Regulation of the IFN system is then of great importance to COVID-19 outcomes: under activation in the early stages allows SARS-CoV-2 into the body undetected, while overactivation in later stages results in serious damage to the host.

These inflammatory responses can cause further damage by contributing to thromboinflammation, a coagulatory response to systemic inflammation through thrombin generation. On infection, monocytes and subendothelial cells release factor VIIa and factor Xa. Leukocytes, endothelial cells, and platelets release proinflammatory cytokines and procoagulant microparticles, promoting increased leukocyte adhesion and decreased vasculo-protective molecules. These result in NETosis—the activation and release of neutrophil extracellular traps that recruit yet more inflammatory leukocytes and cytokines (Figure 2). Eventually this can lead to a loss of homeostasis and damaged microvasculature, called disseminated intravascular coagulation [38]. Thus, several positive feedback loops encourage the release of proinflammatory cytokines and can result in serious damage to the host.

## 3. Adaptive Immune Response during COVID

### 3.1. Adaptive Immune Response Time to COVID-19

The adaptive immune response takes at least five days to take effect [7]. For COVID-19 it was found that antibodies are detectable approximately six days after RT-PCR detection of viral infection [39]. Milder cases of COVID-19 generally take longer to show detectable levels of antibodies, with some being up to 28 days post RT-PCR confirmation of viral infection [39]. The deadly cytokine storm that some severe cases of COVID-19 patients had was generally triggered about one week post infection [39]. Finally, it was found that antibodies last longer in patients who had severe cases of COVID-19, generally lasting at least six months, while more mild cases had antibody levels that faded by 2–4 months post viral clearance [39].

### 3.2. Antibodies for COVID-19

Antibody responses to COVID-19 are essential for viral clearance. Antibody maturation increases the body’s ability to defend against SARS-CoV-2 infections [40,41,42]. When tested several months after infection, serum was discovered to have low antibody levels specific for single variants of SARS-CoV-2, but high levels of antibodies capable of recognizing the common epitope of several variants [40]. Additionally, even more antibody variance was prompted by repeated exposure to slightly different variants of the coronavirus [40].

Another study using plasma taken from 1–10 months post SARS-CoV-2 infection showed that initially the antibodies only protected well against the original variant that the patient was infected with, but plasma taken further from initial infection showed higher protection against variants of concern (VOCs). This indicates that although the total amount of antibody in serum may be declining, the protection offered against different variants of SARS-CoV-2 infection may not be declining much, if at all [42].

However, another study showed that protection offered from vaccines against different variants of SARS-CoV-2 was not guaranteed [43]. This study found that a relatively small number of mutations could lead to an escape from immune neutralization. However, it is important to keep in mind that this study was performed only a few weeks after vaccination, not giving the antibodies as much time to mature. Additionally, not all participants received the full schedule of vaccine doses that is recommended, and only half of the VOCs tested were even able to partially escape neutralization from the vaccine induced humoral immunity [43]. Protection against VOCs of SARS-CoV-2 is increased in the months following infections as antibody maturation allows for a broader immune response against more variants of the infection. This is illustrated in Table 3, which shows how immune response in the months following an infection with SARS-CoV-2 is increased, especially when the initial infection was severe.

Immune response between vaccines and natural infection differs in two main ways [40,41,42]. First, the immune response to a natural infection tends to be broader, as the infection itself may consist of several slightly different variants. This also assists antibody maturation in the months following infection. The second difference is the strength of natural immunity over time, as compared to the vaccine response when responding to new variants. Because the antibodies from a natural infection are much more prone to undergo antibody maturation with slightly different binding sites, the natural immune response may have a better response to fighting new variants. This is specifically shown in the 3–6 months range after recovery from a moderate illness (Table 3).

Finally, one more hopeful study showed that following receipt of a third dose of the BNT162b2 mRNA COVID-19 vaccine, the likelihood of a severe outcome from any of the variants of COVID-19 was highly reduced [44]. However, it is important to note that this antibody response to the vaccine is not uniform in all patient populations. For example, immunosuppressed patients such as those with chronic immunological diseases, dialysis, transplant patients, and patients with hematological malignances have a lower seroconversion rate [45,46].

### 3.3. CD8^+^ & CD4^+^ T-Cell Response for COVID-19

One study examined the effect prior COVID-19 infection has on CD4^+^ and CD8^+^ T-Cell responses to COVID-19 vaccination [47]. Regardless of infection status, upon vaccination, CD4^+^ T-Cells immediately rose to higher levels. However, the cytotoxic T-cells only rose to high levels after a single dose for individuals that already had a SARS-CoV-2 infection, while those who were naive only had a boost in cytotoxic T-Cells following a second dose several weeks after the initial vaccination [47]. This provides evidence of strong memory T-cells and prolonged immunity even following the loss of serum antibodies for COVID-19 [48].

The importance of memory B and T cells in prolonged immunity to COVID was further shown in a study by Cox et al. [49]. There, it was shown that, although antibodies produced in response to mild COVID-19 infection only have a 21-day half-life, the memory T and B cells formed maintain their protective capabilities from similar strains of SARS-CoV-2 for much longer. Research from similar human coronaviruses has shown that memory T and B cell responses last for several years after infection, and that memory T cells are essential in the quick response of B cells in antibody making and in the formation of cytotoxic T cells [49].

### 3.4. Natural Killer Cells’ Response to COVID-19

NK cells play a key role in the immune response to COVID-19. Natural killer cells work to attack and lyse infected body cells containing COVID-19 viral RNA and thereby allow the antigens into the bloodstream to be detected by other parts of the adaptive immune system. This forms a targeted response to the COVID-19 viral RNA, spike protein, and other antigens. In a recent study, the immune cell count that correlated the most with survival rate and least with severity of disease was the NK cell count [50].

## 4. Immune Clearance of COVID-19

### 4.1. Immune Cells through Infection

The efficiency of viral clearance for COVID-19 is significantly affected by CD4^+^ and CD8^+^ T cells. Virus-specific CD8^+^ T cells have been associated with better outcomes in COVID-19 infections, as they kill infected cells with their cytotoxins. CD8^+^ T cells are crucial for clearance of many viral infections [51]. COVID-19 virus clearance requires both adaptive and innate immune responses, but in innate immunity, macrophages can contribute to disease progression. A significant amount of the cytokine IL-6 is produced by macrophages during COVID-19, suggesting they may contribute to excessive inflammation [52]. The innate immune system dominates the early immune responses to viruses. Within this early response, many leukocytes are secreted including neutrophils, monocytes, plasmacytoid dendritic cells (pDCs), and NK cells. Once an adaptive immune response is triggered, T and B cells become critical for viral clearance which develops over days to weeks [53].

Among the factors that may impede viral clearance of COVID-19 are decreases in the number of circulating NK cells, Th1 CD4^+^ T cells, pDCs, phagocytic neutrophils and monocytes, as well as the immunomodulatory properties of progesterone, which is elevated in pregnancy. Factors that may exacerbate COVID-19 morbidity through hyperinflammatory states include increases in the complement system, increases in TLR-1 and TLR-7, and increased pro-inflammatory cytokines such as IL-6 and TNFα. Increases in complement system activity are linked to greater lung injury [54]. It has been well studied that during viral infections, a decrease in Th1 reactivity can result in less efficient clearance of infected cells. However, an overt Th1 and Th2 response to COVID-19 has been implicated in the pathogenesis of severe COVID-19 [55].

### 4.2. Early Infection vs. Late Infection

Another factor that remains unclear in viral clearance of COVID-19 is ACE2. ACE2 is a key component of the renin-angiotensin system, which cleaves angiotensin II to generate ang1-7. Increases in vascular permeability and immune cell infiltration is associated with lung edema due to angiotensin II accumulation in the lungs, and the reduction of ACE2 expression has contributed to acute lung failure through modulation of the renin-angiotensin system [56]. It has been reported that the expression levels of ACE2 played an important role in determining the outcomes of COVID-19 infections. During the early stage, lower levels of ACE2 in the lung is beneficial for the host to control viral transmission and replication. However, it is possible that if not enough ACE2 is present for a prolonged period, the resulting lack of ACE2 could cause angiotensin II to be converted less effectively to ang1-7. Consequently, the accumulated angiotensin II might cause increased immune activity and eventually lung disease [56].

In order to clear an infection effectively, patients must possess CD8^+^ effector T cells that can kill virally infected cells, as well as CD4^+^ T cells that can enhance the CD8^+^ and B cell responses. However, cytokine release by T cells can also contribute to severe tissue inflammation and toxicity, resulting in mortality [57]. While cytokines are critical for the innate immune response and successful clearance of viral infections, their release must be controlled to prevent systemic cytokine storm and harmful inflammation during COVID-19 infection [58]. Therefore, immune checkpoints are significant because they help regulate effector T cell responses. If short term viral clearance is achieved, the majority of virus-specific T cells undergo apoptosis, but for long term viral clearance, the retention of the virus-specific memory T cell population is necessary [57].

### 4.3. Factors Necessary for Viral Clearence

It has been shown that humoral immunity is not vital in clearing acute COVID-19 if there are sufficient amounts of CD8^+^ T cells, and that the major role of CD4^+^ T cells in the clearance of COVID-19 is to instruct humoral immunity with a much lighter role in amplifying cellular immunity [59]. In studies with B cell-deficient mice, antibodies alone were successful in clearing COVID-19, albeit slower than when paired with a fully competent adaptive immune response. However, viral clearance was impossible if neither CD4^+^ nor CD8^+^ T cells were present. In line with these findings, antigen-specific CD4^+^ T cell profiling of acute and convalescent COVID-19 patients indicated that circulating T follicular helper cells play a role in reduced disease severity, further proving that antibody promotion of CD4^+^ clearance is important [59]. Moreover, studies on COVID-19 patients showed that antigen-specific CD4^+^ T cells could be detected as early as 2 to 4 days following symptom onset, and this early detection was associated with improved outcomes. It appears that both humoral and cellular immunity contribute to COVID-19 clearance during primary infection, which is in agreement with patient studies showing a connection between clinical outcomes and a robust coordinated adaptive response in which CD4^+^ T cells, CD8^+^ T cells, and antibodies are often required [59].

Individuals with moderate COVID-19 showed evidence of productive innate and adaptive immunity, characterized by early transient increases in monocytes and NK cells, followed by sustained increases in memory T and B cells. Individuals with severe disease have exhibited symptoms suggestive of an immune response dysregulated by delayed and prolonged increases in Tfh cells, HLA-DRlo monocytes, and activated CD8^+^ T cells [53].

Adequate T cell homeostasis is required for successful viral clearance and clinical improvement [57]. Chronic viral infections have been cleared with the use of treatments aimed at reducing T cell exhaustion or death. Studies have shown that both IL-7, which increases T cell self-renewal, and blocking of the inhibitory immunoreceptor-mediated interaction that suppresses T cell proliferation, such as PD-1/PD-L1, can promote antiviral immunity [57]. CD4^+^ T cells specific to COVID-19 were rapidly induced in patients with acute COVID-19 and resulted in accelerated viral clearance [60].

The role of leukomonocytes in COVID-19 viral clearance is not yet clearly defined, although previous studies have pointed out that suboptimal T cell and B cell responses can slow down viral clearance in patients infected with MERS-CoV and SARS-CoV. Lymphopenia was common in 25 COVID-19 patients, but after 2 weeks, the patients that cleared their infections presented restored numbers of CD3^+^, CD4^+^, CD8^+^ T cells and B cells. The recovered patients had a higher count of leukomonocytes [61].

In addition to T cell homeostasis, the cytolytic effects of NK cell function play an important role in COVID-19 clearance. NK cells that expressed receptor DNAM1 have been linked to more rapid recovery [62]. As NK cells play a key role in the innate immune system’s viral clearance, a decrease in their populations may lead to a reduction in COVID-19 viral clearance [61].

### 4.4. Viral Clearance of Different SARS-CoV-2 Strains

SARS-CoV-2 has evolved multiple variants such as Alpha, Beta, Gamma, Delta, and Omicron, which have proliferated amongst the world population over the last two years. Mutations accumulated in different SARS-CoV-2 variants dictate the variant’s tissue tropism [63]. For example, the Omicron variant has a higher affinity for human ACE2 compared to previous variants. This is due to a significant number of mutations in the virus’s receptor-binding domain (RBD). The Omicron mutations involved in this high binding affinity with human ACE2 include Q493R, N501Y, S371L, S373P, S375F, Q498R, and T478K [64]. With these mutations, the entirety of Omicron’s spike protein and RBD include a high proportion of hydrophobic amino acids such as leucine and phenylalanine. These amino acids are located within the protein’s core and increase the structural stability. This increases Omicron’s affinity to ACE2 and explain Omicron’s high transmission rates [64,65]. This unique feature of Omicron may suggest that, clinically, greater emphasis should be placed on the initial phase of disease where the virus replicates consistently. However, more research is needed on this topic.

The clinical significance of different strain’s tropism is also important when considering the Delta variant. The Delta variant, with a tropism ideal for the lower respiratory tract, has emerged as a deadly variant compared to the variants with tropism for the upper respiratory tract [66,67,68]. Infection-induced inflammation in the lower respiratory tract interferes directly with lung function and is life-threatening [69]. Thus, for patients infected with the Delta variant, additional focus should be directed to combating the hyperinflammatory stage of disease [70].

### 4.5. Immune Exhaustion

Hypercytokinemia is a severe innate immune response to COVID-19 infection, and contributes to immune exhaustion during the adaptive immune response [71]. Mechanistically, severe disease is caused when excessive cytokine production leads to a state of lymphocyte exhaustion, potentially opening the door to opportunistic infections [72]. Additionally, it has been shown that COVID-19 is capable of ACE2-independent infection of T lymphocytes, further contributing to lymphocytopenia and T cell exhaustion [73]. There is a strong correlation between late-stage lymphocytopenia and more severe cases of COVID-19, as approximately 83% of patients wth COVID-19 display lymphocytopenia upon hospital admission [74].

The ability of COVID-19 to infect T-cells and induce their dysfunction may also allow the virus to circumvent immune checkpoints and further promote T cell overuse and exhaustion. This is similar to the way cancerous cells and other viruses such as human immunodeficiency virus (HIV) cause mortality by triggering overactivity and subsequent exhaustion of the host immune system. Immune checkpoints are points in the cycle of immune regulation that are influencable primarily by cell-surface regulators [75]. For example, deficiency in the P-selectin glycoprotein ligand-1 (PSGL-1) adhesion molecule has been correlated with better T cell survival and improved viral clearance in mice [76]. Certain melanoma cells that constitutively express high amounts of similar cell surface ligands may likely trigger T cell exhaustion this way.

The interplay between COVID-19 infection and immune checkpoints has led researchers to investigate immune checkpoint inhibitor (ICI) therapy as a potential route of treatment for COVID-19 infection [77]. ICI therapy is already used widely as a cancer treatment. Current data indicates that ICI therapy is safe to continue during COVID-19 infection. The success of ICI therapy at improving outcomes for patients with COVID-19, however, seems predicated on the timeline of T-cell differentiation during infection. For example, if ICI treatment is used prior to or early during a COVID-19 infection, it enables activation of effector T cell functions that can benefit anti-COVID-19 immune response. When administered later, it is possible that ICI treatments can contribute to the hyperinflammatory response. It is therefore recommended by researchers to utilize regular and differentiated screening of lymphocytes to determine when it is safe to utilize ICI treatment [78].

### 4.6. Tissue-Dependent Responses to COVID-19

COVID-19 pathophysiology is tissue-dependent, and antibody and cytokine responses differ between plasma and nasopharyngeal samples. One study found that, while a majority of COVID-19 patients underwent seroconversion, spike-specific antibody presence in nasopharyngeal secretions was less common [79]. Additionally, the correlation between local and systemic spike-specific antibody response and neutralization activity was poor, suggesting tissue-dependent regulation of these processes.

Cytokine response was also found to differ between plasma and nasopharyngeal samples. Plasma samples of COVID-19 patients showed significant differences in the presence of 13 cytokines as compared to healthy individuals, while nasopharyngeal samples showed differences in seven cytokines, five of which were different than the cytokines identified in the plasma group [79]. Cytokine response also appears to be dependent on disease severity. In systemic samples, 10 circulating cytokines were found to have significant differences between critical and non-critical COVID patients, while 13 cytokines, mostly non-overlapping with those 10, were found to have differences between critical and non-critical patients in nasopharyngeal samples [79]. These findings suggest that, as with spike-specific antibody response, cytokine response is tissue-dependent.

Differences between systemic and local responses were also found when analyzing viral load. While increased viral load in plasma samples correlated with increased disease severity, nasopharyngeal viral load was found to be independent of disease severity [79].

The differences between local and systemic responses to COVID-19 may suggest that, beyond curating treatment to the different phases of disease, clinicians might be able to develop treatments specific for the different body regions impacted by COVID-19. However, more research is needed to elucidate the full efficacy of this approach.

### 4.7. Future Research

As shown in numerous studies, the role of T cell performance in COVID-19 is crucial to viral clearance [53,57,59]. Transfusions of CD4^+^ and CD8^+^ T cells may prove very rewarding in clearing COVID-19. Additionally, Th1 levels are important to regulate in a clinical setting because too high or too low levels of Th1 may contribute to COVID-19 pathogenesis. To decrease morbidity due to hyperinflammatory states from increased expression of TLR-1, TLR-7, and increased pro-inflammatory cytokines such as IL-6 and TNFα, these factors may need monitoring and even partial inhibition. Many factors of COVID-19 remain unclear and further studies into specific immune responses would be helpful in finding effective treatments for viral clearance. Additionally, further research is needed to further elucidate the differences between SARS-CoV-2 variants as they relate to immunotherapy and the multi-phasic nature of COVID-19.

## 5. Post-Acute Sequelae of COVID-19

Shortly after the beginning of the COVID-19 pandemic, a phenomenon known as “long-COVID,” or Post-Acute Sequelae of COVID-19 (PASC), appeared to medical professionals [80]. On average, COVID-19 symptoms are resolved in 1–4 weeks, making patients with symptoms lasting longer than 28 days candidates for PASC diagnosis. Many studies have found that a large number of COVID patients report symptoms lasting longer than 28 days.

One study performed in early 2021 based out of the University of Washington surveyed 177 COVID-19 positive individuals between 3 and 9 months after symptom onset [80]. It was found that about 30% of outpatients reported persistent symptoms, corroborating an earlier study that found that 36% of outpatients had not returned to baseline health by 14 to 21 days after infection [81].

These early studies were limited by small population sizes. A later international study analyzing self-reported COVID-19 symptoms from 4182 patients found that 13.3% of participants reported symptoms lasting 28 days or longer; 4.5% reported symptoms lasting longer than 56 days; and 2.6% reported symptoms longer than 84 days [13]. The same study found that PASC was characterized primarily by symptoms including fatigue, headache, dyspnea, and anosmia; and that early disease features were generally predictive of the duration of symptoms.

Overall, these studies support the belief that PASC symptoms were linked specifically to past COVID-19 diagnoses. Although they disagree somewhat about the proportion of COVID-19 patients that develop PASC symptoms, the experience of “long-COVID” is prevalent in at least some notable percentage of COVID-19 patients. Since then, many further retrospective studies have been published that highlight a high incidence of PASC symptoms in about one third of COVID-19 patients (see Table 4).

### 5.1. Potential Causes

The proximal cause of PASC is unknown and studies disagree about the most likely triggers. Some studies suggest that a cytokine storm of inflammatory cytokines causes tissue damage to major organs, causing PASC symptoms. On the other hand, one study performed on patients with pneumonia secondary to COVID-19 infection suggests that persistent symptoms may be attributable to “biopsychosocial” effects of COVID-19 [85]. Still, many studies dispute the possibility that PASC has a single proximal cause and instead attribute the range of PASC symptoms to a myriad of differing pathological traits of the virus [86].

### 5.2. The Cytokine Storm and Hyperinflammation

The phenomenon of the cytokine storm, or hypercytokinemia, is not unique to COVID-19 infections, although it is a commonly cited cause of COVID-related mortality [87,88]. In essence, hypercytokinemia is characterized by three markers [89]:

1. Perpetuated activation of lymphocytes and macrophages causing immune dysregulation;
2. Large secretions of cytokines caused by such perpetuated activation;
3. Overwhelming systemic inflammation and multi-organ failure with high mortality.

Early in the pandemic, high levels of inflammatory cytokines were observed in patients with poor outcomes. The upregulation of IL-6, in particular, has been correlated with poor COVID-19 prognosis in a large study of 1,473 patients [90]. In fact, serum concentrations of IL-6 above a threshold range from 35 to 80 pg/mL have been correlated with a substantially higher likelihood of mortality [91,92]. Intensive care unit (ICU) patients were also found to have higher plasma concentrations of the proinflammatory cytokines IL-2, IL-7, IL-10, GSCF, IP-10, MCP1, MIP1A, and TNFα, compared to non-ICU patients [11]. This, coupled with a spike in other inflammatory markers such as enhanced concentrations of C-reactive protein, have led many researchers to conclude that COVID-19 mortality is strongly correlated with hyperinflammation [93].

Additionally, many PASC symptoms are consistent with inflammatory organ damage. For example, one study by the CDC surveyed more than 900 hospitals and found that the risk of myocarditis for COVID-19 patients was, on average, 15.7 times higher than for patients without COVID-19 [94]. Another study conducted in Germany on 100 individuals recovering from COVID-19 (median 71 days after diagnosis) found that 71% of these patients had elevated levels of troponin in their heart tissue [95]. The same study found that 78% of patients had abnormal results from cardiovascular magnetic resonance imaging.

Identifying hypercytokinemia as the proximate cause of PASC explains the myriad of symptoms associated with Long-COVID. Since cytokines are prevalent in circulation, they are able to access many different organ systems. As a result, a hypercytokinemia-related prognosis may result in a variety of symptoms depending on the patient’s own physiology. Supportive of this is a relatively rare complication of COVID-19 infection; multisystem inflammatory syndrome (MIS). COVID-related MIS is characterized by a cytokine storm secondary to COVID-19 exposure [96].

### 5.3. Myalgic Encephalomyelitis

**Figure 3:**
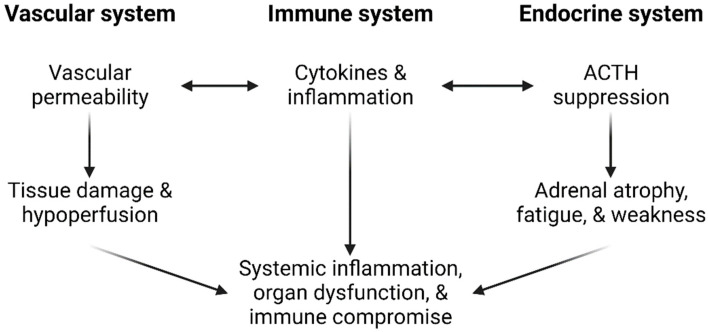
Vascular and endocrine “vicious cycles” associated with myalgic encephalomyelitis.

A growing body of evidence suggests that the phenomenon of Long-COVID may be closely related to another condition called myalgic encephalomyelitis (ME). Known also as chronic fatigue syndrome (CFS), ME is a debilitating illness that has been well documented to affect millions worldwide. Viral infections are one of the leading known causes of ME [97]. Although the exact pathology of ME is poorly understood, it has been proposed that the mechanism involves many different body systems in response to the stress of severe infection [98]. Particularly well documented are the interlinked effects on the vascular system, intestines, endocrine axes, and thyroid hormone function (Figure 3).

Research conducted on these interlinkages highlights the role of cytokines and inflammation in creating “vicious cycles” that may explain the chronic nature of ME. For example, vascular permeability is subject to an IL-6 mediated positive feedback loop characteristic of septic hypoperfusion [99]. Similarly, pituitary activity is suppressed by cytokine activity. Among other effects, cytokines suppress the pituitary release of adrenocorticotropic hormone (ACTH) [100]. Since ACTH stimulates adrenal function, prolonged cytokine activity on the pituitary results in excessive inflammatory responses [101].

Fundamentally, myalgic encephalomyelitis is a multi-organ system condition perpetuated by positive feedback loops involving cytokine-mediated inflammatory reactions. This description draws close parallels with post-COVID MIS. The similarities of PASC to ME are striking, with a significant overlap in symptoms including lasting fatigue, unrefreshing sleep, and brain fog. Additionally, it has been reported that arrhythmic diseases may present during and after SARS-CoV-2 infection [102]. Further strengthening this comparison is an open letter published in the British Medical Journal in August 2021 highlighting that approximately 25% of COVID patients developed ongoing symptoms that meet the diagnostic criteria for ME [103].

### 5.4. Other Explanations for PASC

Another hypothesis is that hypercytokinemia cannot be the driving cause of PASC since a cytokine storm may be necessary for viral clearance. This is substantiated by the fact that IL-6 levels are lower in COVID patients than in other inflammatory conditions such as acute respiratory distress syndrome or bacterial sepsis [104]. Additionally, hypercytokinemia—as indicated by elevated levels of IFN-γ, IL-6, IL-1 and TNF-α—is a documented symptom of other viral conditions, including H1N1 influenza [105]. In fact, the H1N1 cytokine storm was associated with inflammatory pulmonary compromise and mortality, similar to COVID-19 pathology [105]. The key difference is that recovered H1N1 patients reported persisting symptoms infrequently compared with that of recovered COVID-19 patients [106].

Another hypothesis disputes the designation of PASC as a condition at all. One article attributed the phenomenon of Long-COVID to the “biopsychosocial” effects of COVID-19 [85]. This study found that although 86% of 134 COVID-19 pneumonia patients discharged from the hospital reported residual symptoms on follow up, none of these patients had detectable radiographic abnormalities at that time. Essentially, these researchers concluded that Long-COVID was a psychosomatic condition.

These explanations are limited in their scope. The study concluding that PASC symptoms are due to biopsychosocial effects of the COVID-19 pandemic made that assertion despite the fact that only COVID-19 pneumonia patients were included in the study. As a result, the lack of radiographic abnormalities does not eliminate the possibility of PASC symptoms resulting from other organ system abnormalities. The position that hypercytokinemia is necessary for viral clearance relies upon the assumption that cytokine storms may not both be necessary for clearance and potentially detrimental to patient outcomes. More research is needed in this area to fully elucidate the etiology of PASC.

Most evidence suggests that hypercytokinemia plays a prominent role in an inflammatory reaction resulting in multi-organ damage that has been associated with the Long-COVID phenomenon. ME and COVID-related MIS are strikingly similar, supporting the likelihood that PASC secondary to multisystem organ damage is triggered by mechanisms similar to ME. The correlation is close enough to suggest that PASC should be designated as a particularly virulent subcategory of ME symptoms that are specific to COVID-19 pathology.

## 6. Treatments of COVID-19

### 6.1. Treatments of Acute COVID-19 and Early Phases of Disease

There are several different treatment options for COVID-19, and these fall into four major categories: drug repurposing, monoclonal antibody treatment, new drug development, and symptom management [4].

Drug repurposing, also termed repositioning, is the use of drugs approved to treat one disease for the purpose of treating another [107]. Since drug repurposing utilizes substances which have been thoroughly studied, with well-known pre-clinical, pharmacokinetic, and pharmacodynamic profiles, the drug can be fast-tracked through to phase 3 human clinical trials [108]. This makes the drug discovery and approval process faster, cheaper, and largely more reliable. Remdesivir is one such drug which has been repurposed towards the treatment of COVID-19 [109]. Remdesivir is an antiviral drug originally developed as a treatment for Ebola virus disease and which functions by interfering with viral RNA-dependent RNA polymerase activity [110]. Clinical trials have shown remdesivir to be effective for the treatment of COVID-19, and, although the full efficacy of this treatment is still being investigated, the FDA granted emergency use authorization of remdesivir for patients with severe COVID-19 and most recently expanded use of remdesivir treatment to outpatients with mild-to-moderate COVID-19 disease [111]. Further investigation into the use of remdesivir, as well as of other repurposed drugs such as ivermectin, lopinavir/ritonavir, and chloroquine (hydroxychloroquine) for the treatment for COVID-19 is ongoing [4,112,113].

Another promising type of treatment for COVID-19 involves the use of monoclonal antibodies. Several monoclonal antibodies have already been developed, including those which target the spike protein on the virus and the RBD, which is used by the virus to bind to host ACE2 and enter the host cell [1,114]. These include sotrovimab, bamlanivimab, etesevimab, asiriviamb, and imdevimab, among many others [114]. Monoclonal antibodies may also be used to control the cytokine storm, and these include clazakizumab, siltuximab, levilimab, and adalimumab, among many others [114]. The full efficacy of monoclonal antibody treatment is yet to be fully elucidated, as there are several concerns with this treatment modality. These include production constraints and susceptibility of the treatment to virus mutation [4]. However, with ongoing research and the continual fine tuning of treatment criteria or indications, there is much promise that this will continue being a successful treatment option.

The development of an oral drug that patients may take at home is another avenue showing great promise. The ability to take a drug at home would allow patients to receive treatment at the early stages of infection, and thus reduce the number of hospitalizations and subsequent mortality [4]. In fact, these are the exact results seen in a phase 3 clinical trial of molnupiravir (EIDD-2801), an oral drug produced by Merck which functions similarly to remdesivir by disturbing the activity of viral RNA polymerase [115,116]. Pfizer produces the second class of oral drug which is becoming available in the treatment of COVID-19, called paxlovid (nirmatrelvir-ritonavir) [117]. Paxlovid functions differently than molnupiravir, behaving as a protease inhibitor which disrupts virus replication [118]. These two oral medications will continue being studied intensively as their use becomes more widespread.

Aside from these drugs, patients suffering from acute COVID-19 are treated with anti-inflammatory agents such as steroids and immunosuppressants [119]. This is to reduce the negative effects or hyperinflammation and immune overactivation. Oxygen therapy is also used to reduce the hypoxia often experienced by patients with inflammation in the airways. Similarly, anticoagulants such as enoxaparin, heparin, and apixaban have been used to mitigate the increased degradation of fibrin, consumption of coagulation factors, and thrombocytopenia observed throughout the course of infection [120].

### 6.2. Treatment of Post-Acute Sequelae of COVID-19 and Late Phases of Disease

The likely implication of hypercytokinemia in Long-COVID has led physicians to attempt to treat these conditions with immunosuppressive agents. In particular, Tocilizumab has been used in treatment because of its effects as an IL-6 antagonist [91]. Tocilizumab treatments have proven to improve clinical outcomes for patients with severe COVID-19 [14,90].

The success of tocilizumab therapy must be tempered by acknowledging the dangers associated with immunosuppressive treatments. In weakening the immune response to COVID-19, immunosuppressive treatments may open the door to additional infection. As a result, this treatment is currently only approved for patients with severe cases of COVID-19 in which recovery is unlikely without extreme interventions.

Convalescent plasma therapy (CPT) is another treatment that has proven successful in the management of severe COVID-19 cases [15]. In combination with tocilizumab therapy, CPT was found to reduce plasma IL-6 levels much faster than either therapy on its own.

One of the downsides to the similarity of ME and PASC pathology is that there are likely no treatment possibilities other than symptom management. Instead, emphasis should be placed on treating seriously ill COVID patients with preventative immunosuppressive therapies such as tocilizumab in the most severe cases, or less invasive CPT treatments when available. Unfortunately, however, many non-ICU patients that would not be eligible for tocilizumab treatment report PASC symptoms.

## 7. Conclusions

In conclusion, our review demonstrates that COVID-19 is a multi-phasic disease composed of three stages: viral replication, immune hyperactivation, and PASC. This has been paramount in the tailoring of treatment options for patients with COVID-19, as treatments for the early stages of the disease involve antivirals and monoclonal antibodies, while latter stages of the disease are treated with heparins and immunosuppressants. As more treatments are developed and brought into clinical practice, a continued emphasis on the multiphasic nature of COVID-19 will remain of paramount importance. Our review also describes areas where future research is needed and will become fundamental to understanding the body’s immune response to COVID-19.

## Figures and Tables

**Table 1:** Phases of COVID-19 infection and the corresponding treatment approach.

**Infection Phase**	**Clinical Presentation**	**Treatment Approach**	**Sources**
Viral replication	Upper respiratory tract infection, fever, muscle fatigue, pain	Antiviral agents are used to decrease viral load, transmission, and prevent progression to the next phases of the disease	[10,11,12]
Immune hyperactivation	Dyspnea, pneumonia, vasculopathy, acute cardiac and renal damage, sepsis, secondary infections	Monoclonal antibodies, anti-coagulants, immunosuppressants, oxygen, antiviral drugs	
Post-Acute Sequelae of COVID-19	Fatigue, headache, dyspnea, and anosmia	Immunosuppressants, convalescent plasma therapy	[13,14,15]

**Table 2:** TLR detection of SARS-CoV-2.

Toll-Like Receptor	Interaction	Source
TLR1	Binds SARS-CoV-2 spike protein	[28]
TLR2	Interacts with viral envelope protein to induce pro-inflammatory response	[26]
TLR3	Recognizes double-stranded RNA and is activated within 24 h of SARS-CoV-2 infection	[27]
TLR4	Binds SARS-CoV-2 spike protein	[28]
TLR6	Binds SARS-CoV-2 spike protein	[28]
TLR7	Detects single-stranded RNA	[27,29,30]

**Table 3:** Protection against variants of concern (VOCs) of SARS-CoV-2 from natural immunity.

Severity of Infection	Time Post Infection	Protection Level Against VOCs *	Sources
Severe	1–3 months	Low to high	[40,41,42]
	4–6 months	Medium to high	
	6+ months	High	
Mild	1–3 months	Low to medium	
	4–6 months	Medium to high	
	6+ months	Medium to high	

* As measured in affinity assay by Muecksch et al. and neutralization assay by Moriyama et al. [41,42].

**Table 4:** Studies reporting long-COVID data and the percent of outpatients with persisting symptoms.

Date	Study Size	Mean Population Age (Standard Deviation, When Provided)	Gender	Percent of Outpatients with Persisting Symptoms	Source
May 2020	350	Median: 43 *	F: 53% M: 47%	36% (14–21 days)	[81]
March 2021	177	48	F: 57.1% M: 42.9%	32% (median: 169 days)	[80]
April 2021	4182	45.97 (15.8)	F: 71.5% M: 28.5%	13.3% (28+ days)	[13]
July 2020	143	56.5 (14.6)	F: 37% M: 63%	87.4% (Mean 60.3 days [SD: 13.6])	[82]
Sep 2021	106,578	39.4 (18.4)	F: 58.4% M: 41.6%	36.55% (90–180 days)	[83]
Feb 2022	5,080,312	See source **	F: 61.2% M: 38.8%	35% (31–150 days)	[84]

* Only the median age is provided in this study; ** Mean age of study participants was not provided in this study. However, study participants were categorized as older or younger than 20 years of age.

## Data Availability

Not applicable.
